# Matrons in Council: VII the Future of the Nursing Profession

**Published:** 1917-09-22

**Authors:** 


### THE MATRONS' AND SISTERS' DEPARTMENT.

### MATRONS IN COUNCIL.

### VII. THE FUTURE OF THE NURSING PROFESSION.

### From the Point of View of an Idealist.

All thinking women of the nursing profession must often give careful thought to what the future of our profession is to be.

It would be advisable, as well as helpful, to take a retrospective view of what has been accomplished, and ask ourselves if the ideals of to-day are of the high order that we read they were of fifty years ago. I had the pleasure of listening to a lecture a short time ago. The lecturer (who is a member of the profession) at one point remarked: Nursing at one time was looked upon as "a vocation," and only those who felt that the call had come specially to them responded and took up their vocation, much in the same spirit that a man might enter for "Holy Orders." After a time it became "a profession," and many ideals which had been existent disappeared. She further remarked she was sadly afraid it was becoming "a trade" and was entered into very rashly by girls who must have a means of livelihood, and was in danger of deterioration from the ideal to the purely commercial. These remarks have often recurred to me, and the thought arises, Have we as a body of nurses failed to live up to the noble example set for us by our pioneers? After careful thought I am sorry to confess that I do not think the aims and ideals of the profession are so high or so pure as those of our predecessors.

At this time, when the whole civilised world is undergoing such an upheaval, one wonders how the nursing profession will emerge from it, and if future generations of nurses will look back upon us with admiration and reverence and say amongst themselves how they must keep the ideals untainted and maintain the same high standard that we of to-day are now setting for them. Let each one of us consider very seriously our respective responsibility in this great question and ask ourselves if we are so conducting our lives that we may be examples not only to those in immediate contact with us, but to those who will follow in our footsteps fifty years hence.

Have you ever lingered in an old-world garden, over which centuries have left their magic spell? Such a garden is mellowed with thoughts of the past. Such a scene produces a reverential awe of what has transpired in the past. So I feel it ought to be with our work; it should be such in the present as shall command regard and gratitude from those who will continue in the work long after we have passed from this sphere of life.

I hear someone say "Yes, but fancy comparing the profession to-day with what it was fifty years ago. Look how we have advanced, think of the methods then employed and how we have improved upon them, the advances in surgery unthought of in those days," etc.

This I grant, but do not let us become egotistical, remember that improvements are going on, not only in our profession, but in every section under the sun. Just so shall improvements be carried out in the future, and as the methods of yesterday appear to be somewhat crude to us of to-day, so surely will our methods appear crude to those who will be of to-morrow.

Material changes must come about in the natural course of events, but the ideals, I am afraid, are not being upheld in a manner worthy of those who carried the standard before us. Discussing this point with a lady who has had many years' experience both of the work and the world, she maintained that the standard of nursing was lowered when the profession seceded from the Church, and until we get back to the Church we shall not recover the ideals which for the time being are apparently lost sight of. I do not think it necessary that we must return to the fold of the Church to recover our hold of the ideals of our profession. But it certainly appears to be imperative that some serious self-analysis and questioning should take place. All nurses capable of deep thought will not be satisfied to let the present state of affairs continue.

Where, then, lies the remedy? There must be a remedy somewhere, and it must not only be found but acted upon diligently. As matron of a training-school for nurses, I believe that a very great part of the remedy lies in the hands of matrons. To illustrate what I desire to impress upon any who may read this, may I quote a very old adage, "The hand that rocks the cradle rules the world"? We know with a child the mother's influence is the first to affect its career and, all through life, holds sway longer than any other, and in every instance where the mother's influence is good and pure it never loses its grip.

So with the nursing profession. Only get a good, solid, and noble foundation, and wherever the nurse goes in the future she will never lose hold of the ideals taught her during the kindergarten stage of her career. A matron accepts responsibility not only that the nurses who acquire their training under her guidance may be turned abroad as perfect nurses, but for the time being accepts a moral responsibility towards them, and aspires not only to turn them out as good nurses, but good, conscientious, and noble women. The matron's task is no easy one, and to hold the reins of any hospital with a firm, just grip will tax the resources of the most capable woman.

Never have matrons and nurses had such an opportunity for noble self-sacrificing work as at this present time, and we must all do our utmost to uphold the highest and best traditions of the profession and leave a worthy example to those who come after.

☛ Previous articles appeared in The Hospital of August 4, 11, 18, 25, and September 1 and 15.

